# Towards digital organized crime and digital sociology of organized crime

**DOI:** 10.1007/s12117-022-09457-y

**Published:** 2022-05-30

**Authors:** Andrea Di Nicola

**Affiliations:** Department Faculty of Law (University of Trento) and Centre of Security and Crime Sciences, University of Trento - University of Verona, Via G. Verdi, 53, 38122 Trento, Italy

**Keywords:** digital organized crime, digital sociology of organized crime, digital criminal organizing, cybercrime groups, crime groups, organized crime activities, organized cybercrimes, organized crime definitions, digital criminology, cybercriminology

## Abstract

As technology has changed people’s lives, criminal phenomena are also constantly evolving. Today’s digital society is changing the activities of organized crime and organized crime groups. In the digital society, very different organized crime groups coexist with different organizational models: from online cybercrime to traditional organized crime groups to hybrid criminal groups in which humans and machines ‘collaborate’ in new and close ways in networks of human and non-human actors. These criminal groups commit very different organized crime activities, from the most technological to the most traditional, and move from online to offline. They use technology and interact with computers for a variety of purposes, and the distinction between the physical and virtual dimensions of organized crime is increasingly blurred. These radical developments do not seem to be accompanied by a new criminological theoretical interpretive framework, with a definition of organized crime that is able to account for the changes that digital society brings to organized crime and generate modern research hypotheses. This article proposes the concept of digital organized crime and the spectrum theory of digital organized crimes, to be embedded within a current, revised sociological theory of the organization of crime and deviance in digital society (a new theory of digital criminal organizing) and argues that the study of digital organized crime will increasingly require a digital sociology of organized crime. Criminologists are called upon to work in this direction.

## Introduction: digital society and (organized) crime

Since the mid-1900s, profound changes and advances in technologies have driven the emergence of a digital society (for a more detailed historical account, see Deb 2014; Dufva and Dufva 2019; Granieri 2011; Luhmann 2012; Skobelev and Yu 2017; Isaacson 2014). We have moved from mechanical and analog technologies to digital technologies that define our everyday lives today. The digital revolution began in the late 1980s. The catalyst for this revolution was the birth of the Internet, the World Wide Web, in 1989. Since that year, more and more electronic computers, including personal computers, have connected to the Internet with unstoppable speed. The first browsers, intuitive software for Internet navigation, spread along with increasingly powerful search engines that allow users to easily find the growing information scattered throughout the nodes of the network.

In the early years of this century, those who went on the Internet did so primarily to exchange electronic correspondence, for mailing lists, for e-commerce, and for access to the first popular auction or online sales platforms such as eBay and Amazon. At the same time, online forums and message boards, websites and personal blogs proliferated. The final turn to digitization then occurs when much smaller but more powerful personal computers are joined by faster connections and, finally, in the first decade of this century, cell phones become the accepted means of communication, capable of accessing the Internet and managing complex software applications. The emergence, growth, and consolidation of digital photography and music at the turn of the twentieth and twenty-first centuries fueled this process, as did the arrival and growth of Skype, YouTube, Facebook, Twitter, Instagram, and other social media, which in a few short years have managed to connect billions of real and digital identities worldwide. As a result, billions of people and businesses around the world are now online: they write, take pictures, and publish in real time. They read, they inform, they make decisions. Billions of words and images are left in virtual space, as are hours and hours of videos, music, billions of likes for billions of interactions: terabytes and terabytes of digital information collected, cataloged, tagged with time and place and other specific metatags. These changes are profound because they are leading to new habits, new ways of meeting, interacting with friends and one’s professional network, new ways of working, buying, paying, saving, managing and protecting one's wealth, transferring it, new ways of organizing, seeking and giving professional advice, traveling, taking care of oneself, voting, interacting with institutions, doing business, making art. And this is happening while around the world not only notebooks and desktop computers are increasingly online, but also, with exponential growth, tablets, iPads, Kindles, MP3 players, peripherals of all kinds, and everyday objects such as watches, televisions, household appliances, and even cars, in a transition to the Internet of Things.

The truly innovative element is that the digital technologies of the new millennium exponentially increase the ability to record and collect information of all kinds, as well as that of computation. Modern cameras read and record license plates of cars and trips and report anomalies in real time. Smarter and smarter cities with increasingly sophisticated sensors collect information of all kinds about residents and tourists. Businesses store information about customer habits and organize themselves based on the knowledge they gain from this digital legacy. Mobile and travel data are mines in the hands of mobile companies. Google Maps shows us the fastest route, processing millions of pieces of data about other drivers and their movements. Latest generation algorithms suggest us what to read, what to buy, where to go, they find us photos similar to the ones we like, they suggest new interests and new contacts. This is because the digital society is also a Big Data society: an ever-increasing amount of data on a previously unimaginable scale, generated by our daily interactions with network technologies, is automatically collected and stored, and it is becoming more diverse, faster, more valuable, and more reliable (Mayer-Schoenberg and Cuckier 2013). Data from which with increasingly powerful computers, artificial intelligence, machine learning, and neural networks it is possible to derive knowledge, correlations, and predictions useful for supporting decisions, actions, and interventions in all kinds of domains, whether personal or institutional.

Today, life is also digital, as the line between the virtual and the real becomes increasingly blurred. Negroponte wrote with clear foresight more than twenty-five years ago in his seminal book Being Digital that «*[t]he change from atoms to bits is irrevocable and unstoppable*» (1995: 4): for the author, everything that can be digitized will be digitized. For Negroponte, digital is a concept that can be related to a digital culture. When he speaks of digital life, he is not just referring to a philosophical, mathematical system or to the technological aspects based on a binary structure, but to the profound impact that digital technologies have on our society. For example, he writes, «*digital life will be less and less dependent on being in a particular place at a particular time*» (1995: 165).

One of the seminal works in digital sociology, that of Deborah Lupton (2015), begins with an introduction titled “Life is Digital.” We live in a digital society where technology has radically changed, and continues to change, the world in which we live. For Lupton, digital technologies are more and more an integral part of many people’s daily lives and social life is now taking shape through them. To assert that society is digital is to affirm that concepts of society and culture cannot be fully understood today without acknowledging that individuals, relationships, and social institutions are shaped by both software and hardware devices. Today’s social is increasingly implemented through digital technology, which is an integral part of social networks as well as social institutions such as the family, the workplace, the education system, the health care system, the mass media, and the economy.

The concept of digital society helps us understand how digital technologies are reshaping modern society. Anthropologists Horst and Miller (2013: 4) believe that digital technologies do not make us less human, less authentic, but are an integral part of what it means to be human today. This new way of being human also implies a strong interaction between the human and the nonhuman. In the Internet of Things, human and non-human actors intermingle, interact, and shape each other in a close relationship that is reshaping society. This conclusion is supported by the stream of Science and Technologies Studies (STS) that has been dominated in recent years by Actor-Network Theory (Callon 1986, 1999; Latour 1987, 2005), an approach that emphasizes the role and agency of nonhuman actors in defining human actors. Human actors would become increasingly enmeshed in networks of human and nonhuman actors that can no longer be considered separately. The concept used to describe the hybrid phenomena created by the interaction between human and nonhuman actors is that of sociotechnical or socio-material assemblage: fewer and fewer social activities in the world are carried out without the use of material tools, and these tools are no longer merely tools for performing tasks but are increasingly becoming constitutive of people's activities and identities (Haggerty and Ericson 2000; Latour et al. 2012; Hodder 2014; Jarrahia and Sawyerb 2019). Assemblages are the complex social, economic, and technological relationships between humans and nonhumans (Mackenzie and Vurdubakis 2011; Langois and Elmer 2013). Today, humans and technologies coexist and any distinction is purely analytical, recognizing in each case that these entities are interdependent in practice (Orlikowski and Scott 2008: 346). Digital society is often made up of socio-material digital objects—think of comments on social media, browsing history, reviews on TripAdvisor, but also, more generally, any digital data that is the result of decisions made by human actors and the use of digital technologies. The software, the hardware, the people who use them, and the socio-material objects that take shape bring to life the assemblages we have been talking about, and are referred to in actor-network theory as “actants” (Latour 1996: 373).

The digital society also has a strong impact on the world of crime. Crimes are changing, e.g., in terms of typologies and *modi operandi*. Criminals change, e.g., in terms of their characteristics, their social (inter)actions, and their relationships with (potential) victims. Where there are new social facts, new habits, new ways to meet, buy, pay, save, protect, transfer assets, new digital identities, new systems for information gathering, self-organization, pleasure and travel, it is only natural that new crimes and new ways to fight crime also emerge. And this is especially true for organized crime, which is committed by organized criminal collectivities that, according to the principles of rationality, always seek to take advantage of new opportunities, maximize their profits, and minimize the risks.

This article is about the relationship between digital society and organized crime. It aims to propose a new criminological interpretive theoretical framework, a definition of organized crime that takes into account the changes that the digital society brings to organized crime activities and organized crime groups, and that is capable of generating more modern research hypotheses. After discussing (in this Introduction: digital society and (organized) crime section) the developments that have led from an analog to a digital world, the concept of digital society, and the studies of digital sociology, this article analyzes the impact of digital society on organized crime activities (Organized criminal activities in the digital society section) and organized crime groups (Criminal socialization and organized crime groups in the digital society section) and proposes the concepts of digital organized crime and the spectrum theory of digital organized crimes (Digital organized crime and the spectrum theory of digital organized crimes section). The thesis is that to understand the new dimension of digital organized crime, a digital sociology of organized crime will be increasingly necessary (Towards a digital sociology of organized crime section): criminologists are called to work in this direction.

## Organized criminal activities in the digital society

There are three types of organized criminal activity related to the new technologies discussed above: cyber-dependent organized crimes, cyber-enabled organized crimes, and cyber-assisted organized crimes. By organized criminal activity in this context we mean serious criminal activities carried out with forms of criminal organization, professionalism, and specialization by organized crime collectivities working together online or offline/online over a prolonged, extended period of time and using communications and information technologies in some way and at various stages.

### Cyber-dependent organized crimes

Cyber-dependent organized crime is high-tech organized crime that can only be committed using computers, computer networks, or other forms of information and communications technology. These are new crimes that have emerged with and are made possible by the digital society: they are the true and ‘pure’ cybercrimes that come to life with the Internet and can only be committed in cyberspace, as Wall (2005) first noted in his work. Without technologies and the Internet, they would not exist. This definition was further refined by McGuire and Dowling (2013), who proposed a subdivision based on how these crimes are committed: unauthorized intrusion into computer networks and disruption or damage to the functionality of computers and networks. The family of illegal intrusions includes hacking, unauthorized access to computer networks, computers, cell phones and tablet devices, exploiting security vulnerabilities mainly to collect personal data or useful information, but also to deface websites and launch DoS or DDoS attacks. The second category, that of disruption and damage, includes malware (malicious software that spreads on computers and interferes with their function and can take various forms, e.g. viruses, worms, Trojans, spyware, ransomware); spam (unsolicited email messages typically sent to countless recipients and often linked to pharmaceutical products or pornography to collect victims’ personal information, including through malware); Denial-of-Service (DoS) (attack that aims to cripple a computer or network by flooding it with unwanted Internet traffic or sending it information that can crash it, making it inaccessible to legitimate users who are deprived of the service or resource they intended to use); Distributed Denial-of-Service (DDos) (a specific DoS that does not originate from a single IP address, but from multiple addresses, often thousands of addresses); Botnets (term for a specific number of Internet-connected computers that make up the botnet, are infected with code, and are under the control of a controller who can conduct illegal activities through them). Many of these organized crime activities can be committed for phishing purposes (Loggen and Leukfeldt 2022). Many others may be conducted as part of “crime-as-a-service” (see, e.g., Hutchings and Clayton 2016). The trade or traffic in hacking tools, hacking services, and the fruits of hacking with malicious intent *«once a varied landscape of discrete, *ad hoc* networks of individuals motivated by ego and notoriety, has now become a burgeoning powerhouse of highly organized groups, often connected with traditional crime groups (e.g., drug cartels, mafias, terrorist cells) and nation-states»* (Ablon et al. 2014: 36; see also Europol 2021; UNODC 2021).

### Cyber-enabled organized crimes

The digital society has given a major boost to a number of traditional crimes whose scale has been magnified by the use of computers, computer networks, or other forms of information and communications technology: these can be named cyber-enabled crimes (McGuire and Dowling 2013). Wall (2005, 2015a) calls them “hybrid cybercrimes,” traditional crimes to which the Internet has opened up entirely new possibilities. By the term “cyber-enabled organized cybercrime,” we mean, for example, computer-related fraud, computer-related identity crimes, online extortion and ransomware, online child sexual abuse and exploitation, and cyberlaundering, which are increasingly committed by various types of organized crime groups (UNODC 2021: 47–95; Europol 2021). Several classic organized crime activities have moved entirely to the Internet and have been facilitated by the digital revolution. In a recent and interesting study, Wang et al. (2021) examined organized Internet-based loan scam and loan sharking activities of traditional organized groups in China at the expense of college students. The world of fraud is among the areas that have benefited most from the advent of the Internet (Button and Cross 2017). Digital society has ‘industrialized’ the scale of fraud (see, e.g., Van Nguyen 2022). Just as modern technology has expanded the ability of businesses to reach larger and more profitable markets, the same is true for fraudsters: frauds that were already committed on a large scale using traditional means of communication such as mail and telephone suddenly became easier, more effective, and more economical when using the Internet and moving in global markets. One example of online organized fraud that is becoming more common internationally and in which African organized crime is particularly active is BEC, Business Email Compromise. In BEC schemes criminals use hacking or social engineering to obtain relevant corporate information via the Internet, which they then use to defraud executives, financial officers, and business owners and induce them to make illicit payments on behalf of the companies they manage (Interpol 2020: 17–19). And, to point out more recent trends related to organized online fraud, particularly online identity fraud, deepfakes will be widely used by organized crime in the coming years (Europol 2022). As Brundage et al. (2018: 19–22) make clear, artificial intelligence can be a powerful tool for criminal purposes because of its ability to amplify existing threats, introduce entirely new threats, or completely alter the characteristics of threats. Deepfakes (Collins 2019; Westerlund 2019) – a word that combines deep learning, an artificial intelligence technique, and fake – are hyper-realistic videos, digitally manipulated by artificial intelligence, of people saying or doing things they never said or did. A deepfake overlays existing videos or images with AI-generated content that resembles a person’s voice or appearance. Deepfakes are “synthetic media”: they imitate people’s faces, movements, and voices with such precision that it is often impossible to distinguish them from reality. Artificial intelligence is also already being used extensively in online organized financial crime. In financial crime (see, e.g., Yeoh 2019), artificial intelligence can be exploited to manipulate the market, fraudulently fix stock prices, and facilitate collusion among multiple players in the market. Artificial intelligence systems can operate autonomously in the service of those who created them to artificially influence stock prices by automatically generating market disruption signals that can truly deceive legitimate actors (Wellman and Rajan 2017: 14). “Pump and Dumb” schemes, where the market prices of a stock are illegally pumped and then deflated to profit from price fluctuations, can be created with social bots that massively disseminate false information to large numbers of potential buyers.

### Cyber-assisted organized crimes

In the digital society, traditional offline crime is facilitated by the use of new technologies. There are complex offline criminal activities that, from a criminal justice perspective, are often interdependent “chains” of crimes that are only facilitated by the use of computers, computer networks, or other forms of information and communication technology. The digital tool is not critical to these forms of organized crime; its role is more incidental, albeit important. In this context, Wall (2005, 2015a) speaks of “traditional crimes” in which the Internet is used as a means of communication or, more generally, to support the organization and its activities.

Wall (2017) specifies that by forms of cyber-assisted crimes we mean those complex criminal activities committed by criminal groups or individuals who use technology to support preexisting criminal operations. The limited literature on cyber-assisted organized crimes focuses precisely on various trafficking activities of organized crime collectivities. Examples include, among others, international research on counterfeit medicine trafficking (Di Nicola et al. 2015; Hall and Antonopoulos 2016; Baratto 2020), smuggling of migrants and asylum seekers (Di Nicola et al. 2017, 2019; Antonopoulos et al. 2020) and trafficking for the purpose of exploitation, particularly sexual exploitation (Di Nicola et al. 2013, 2017; Antonopoulos et al. 2020). Fraser’s (2016) study of human trafficking and social media conducted in twenty-one developing countries also revealed how traffickers rely on new technologies to recruit and communicate with clients, even when they are geographically distant. That digital technologies play a role in modern organized crime activities is also confirmed by research on terrorism or radicalization (Huey 2015; Klausen 2015), the sale of counterfeit products or psychotropic substances (Corazza et al. 2014; Kolliakou et al. 2016; Lange et al. 2010; Walsh 2011), illegal animal trafficking (Siriwat and Nijman 2018; Kitson and Nekaris 2017), and tobacco trade (Munksgaard et al. 2022).

Among the scholars most invested in this direction is Lavorgna (2013, 2014a, b, c, 2015a, b), who has explored how the Internet is used by criminals, particularly organized criminals, to commit transit crimes and how it has changed criminal behaviors and processes by also reconfiguring the relationships between suppliers, intermediaries, and buyers. Lavorgna (2015a, b) has also identified five main types of opportunities that the Internet offers to criminal groups involved in traditional organized criminal activities. In these cases, the network: (i) facilitates communication among criminals and between them and (potential) customers through the use of e-mail, Skype, and other forms of instant messaging (*communicative opportunities*); (ii) improves the efficiency of criminal markets by enabling easy and rapid adjustment of trade to changes in demand (*managerial opportunities*); (iii) facilitates the internal organization of commercial groups (*organizational opportunities*); (iv) enables the expansion of relationships between criminals by creating new trade relationships (*relational opportunities*); (v) also serves as a resource for understanding the needs of potential customers and promoting smuggled products more effectively (*promotional opportunities*).

In considering these opportunities, as well as others also noted in Lavorgna’s work for certain organized crime activities, one should distinguish between those that involve the organization of criminal activity (and we refer to the sociological concept of the “organization of deviance,” that we discuss in this section) and those that involve the organization of criminal groups (which we discuss in the next section, i.e., the relationship between the “organization of deviants” and the digital). The digital society can unleash its effects both on the activities of organized crime by facilitating them and on the way the structures of organized crime groups, the relationships within organized crime groups, and between organized crime groups take shape. To illustrate, the communication opportunities mentioned above (in conjunction with the promotional opportunities) may lead organized criminal groups involved in the entire chain of a particular illicit trade to increasingly offer illicit goods and services online. But these same communication opportunities (combined with organizational and relational opportunities) can also lead stable criminal groups to adopt internal recruitment models (of criminal group members) based on online contacts and relationships, as well as accelerate the spread of organized crime groups with simpler, faster, and more fluid structures. This is because the possibility of cooperation is simplified by communication over the Internet, and it is increasingly easy to maintain close relationships with other criminal organizations and service providers, even if they are physically distant, to whom some of the work can be outsourced, creating “networks of criminal networks.”

Studying the impact of digital society on organized criminal activity as it shifts from the offline to the online world and vice versa is becoming increasingly important, as is studying the impact on the organization of crime collectivities.

## Criminal socialization and organized crime groups in the digital society

According to a quantitative analysis by Weulen et al. (2019), the social ties that develop between perpetrators of high-tech and cybercrime differ from those that form between traditional criminals and are less likely to lead to the sharing of criminal information and common forms of committing cybercrime. Qualitative analyzes have shown that cybercrime offenders share knowledge, criminal opportunity information, and neutralization techniques with online and offline acquaintances and friends (e.g., Holt 2007, 2009; Holt et al. 2012; Hutchings 2014; Hutchings and Clayton 2016). However, as Holt (2007, 2009) also suggests, cybercrime offenders generally work alone but also learn from forums and other online sources. To use Best and Luckenbill’s (1982) categories of deviant organization, cyber-dependent criminals would tend to be of the “colleague” type, often socializing with deviants in their own category but generally operating alone: they hang out together online and offline but do not commit crimes together.

Leukfeldt et al. (2017) confirm that these online social relationships of cybercriminals rarely lead to cybercriminal associations. More specifically, according to the authors, cybercriminal associations emerge in four ways (with the first two accounting for about three-quarters of the total): 1) offline social contacts only; 2) offline social contacts as a base and online forum for recruiting specialists; 3) online forum as a base and offline social contacts for recruiting local criminals; 4) online forums only. The work of Leukfeldt et al. (2017) is part of a line of research focusing on cybercrime groups operating online (see Wall 2014, 2015b, 2017; Broadhurst et al. 2014; Lusthaus 2013). The authors question whether cybercrime groups are organized crime and come to a negative conclusion. Although the authors do not directly define organized crime, they adopt a mafia-centric operational definition that follows a criminological literature that states that for organized crime to exist, in addition to criminal association, commission of serious crimes, duration, and stability over time, other elements such as the use of violence, corruption, and the ability to infiltrate the economy must be present. According to the authors (Leukfeldt et al. 2017: 289), this concept is consistent with those criminological works on organized crime that «*contrast with the very low standards set in policymaking*» for identifying organized crime, according to which the notion of organized crime is nothing more and nothing less «*than just crime that is organized*.» Based on a similar theoretical concept of organized crime, other work also confirms that there is no consistent and robust evidence to draw analogies between cybercrime groups operating exclusively online and organized crime (Lusthaus 2013; Leukfeldt 2015; Lavorgna 2016; Lavorgna and Sergi 2016; Musotto and Wall 2022).

There is no doubt that we do not find all these elements of “traditional”, mafia-type, organized crime in cybercrime groups, or only very rarely. These elements almost never appear in online criminal organizations, just as (it should be emphasized) they are not found in some, albeit stable and reputable, offline criminal organizations. Wall (2015b) points out that the organization of (organized) cybercrime follows a different logic than that of traditional organized crime, which is why Wall calls it a “dis-organized” model: cybercrime groups are based on reputational dynamics, are composed of few individuals, and often do not have a hierarchical control structure. Wall defines them as “assemblages” rather than “organizations.” Unlike traditional criminal organizations, members of an online criminal organization may never have met.

One could also argue that if our way of defining organized crime, which we use as a benchmark, and the characteristics we look for were different, we could probably find them in cybercrime groups. In other words, we can answer the question “*Are online cybercrime groups organized crime?*” differently depending on which criminological definition of organized crime we adopt as a paradigm. For example, if we use paradigms from the criminological literature that define organized crime by recourse to the concepts of “enterprise” (Smith 1975, 1978, 1980; Albanese 1982; Reuter 1985), “network” (Morselli 2009), “governance” (Varese 2010), “criminal organizing” (McIntosh 1975; Best and Luckenbill 1982; Rostami 2016; Rostami et al. 2018), we might arrive at different answers.

In this argument, Broadhurst et al. (2014: 1) claim that «discussions of cybercrime, and of organized crime more generally, are plagued by stereotypes. On the one hand, the image of the lone hacker belies the collective nature of much cybercrime. On the other, conventional definitions of organized crime tend to be out of date, having been overtaken by the evolution of the phenomenon itself».

For the moment, it is more important to emphasize that the above approach considers only one type of crime groups operating in the digital society (online cybercrime groups) and could lead to interpretations that separate the online from the offline dimension of organized crime, concluding that organizing crime and criminals online and organizing crime and criminals offline do not belong to the same genus. Instead, we need a flexible and modern theoretical framework that takes into account the fluidity and speed of digital society and the relevant changes that the Internet and new technologies exert on both the organization of criminals and their criminal activities. A theoretical approach that allows to capture the extreme complexity and nuances of the organization of criminal groups and criminal activities in the digital society, as well as the innovations that the digital society brings to the subjective and objective elements of organized crime.

In analyzing digital society and organized crime, it is necessary to look beyond organized cybercrime groups, in part because, as Bijlenga and Kleemans (2018) note, the distinction between traditional perpetrator offline groups that take advantage of ICT opportunities and criminal groups that operate exclusively online does not seem tenable from a scholarly perspective, as there are many connections between online and offline activities in practice.

McGuire (2012) distinguishes three main types of criminal groups operating in cyberspace, each divided into two subgroups depending on the strength of association between members. These organizational patterns often overlap in very fluid and confusing ways:

1. Type (I) groups, which are mostly “virtual” and are formed through trust relationships based on individuals’ reputations in illegal online activities. They operate essentially online and can be further subdivided into: (1a) “swarms,” described by McGuire as “disorganized organizations [with] common purpose without leadership” (ibid.: 3). Swarms tend to have minimal chains of command and their *modus operandi* is reminiscent of earlier “hacktivist” groups. Indeed, they are most active in ideologically-driven online activities such as hate crimes and political resistance; (1b) “hubs,” which also operate primarily online but, unlike swarms, have a better-defined command structure. They consist of a hub of grassroots criminals, who give instructions to operatives on the periphery. They engage in various illegal activities;
2. type (II) groups that combine online and offline crime and are referred to as “hybrids”. They are divided into: (IIa) “clustered hybrids”, where the organization revolves around a small group of individuals who focus on criminal activities or specific methods and seamlessly switch between online and offline crimes; (IIb) “extended hybrids”, which are much less centralized because they consist of many associates and subgroups, without a clear hierarchical structure;
3. type (III) groups that operate primarily offline but use online technology to facilitate their activities. They are divided into: (IIIa) “hierarchies” or traditional organized crime groups; and (IIIb) “aggregate groups” that are poorly organized, transient, and lack a clearly defined objective (McGuire 2012).

Wall (2017), in attempting to analyze the potential for the growth of new forms of organized crime groups online in a cloud technology environment, also uses the terms cyber-dependent organized crime groups («who commune online and commit crimes online»), cyber-enabled criminal organizations («where new groups of criminals use the internet networks to organize themselves to commit […] crimes […] that they would commit anyway «more locally and in much smaller volumes») and cyber-assisted criminal organizations («where crime groups use technologies to assist their existing operations, including some traditional organized crime groups taking their existing areas of crime business online»).

When we try to understand current developments in digital society, we should also consider new forms of organized crime that seem futuristic, but toward which we are moving more and more. Think of botnets, networks of infected computers controlled by one or more controllers to carry out cyberattacks, and which are increasingly being used for cybercrime. In this context, Broadhurst et al. (2014: 4) state that *«[t]he standard definition of organized crime contained in the UN Palermo Convention, based on the participation of three or more persons acting in concert, does not extend to certain highly sophisticated forms of organization such as the mobilization of robot networks that may be operated by a single person. So-called botnets involve an offender using malicious software to acquire control over a large number of computers (the largest including more than a million separate machines). Even though the individual and institutional custodians of compromised computers may be unwitting participants in a criminal enterprise, some commentators maintain that botnets mobilized by a sole offender should be considered a form of organized crime (*Chang 2012*)».* In this case, we still refer to natural persons: the controller of the botnet and the administrators of the controlled machines. But our perspective, which always refers to organized crime, could also change. In the context of botnets, van der Wagen and Pieters (2015) coined the term “cyborg crime”, i.e. online crime committed by cyborgs, and they mainly propose to use the Actor-Network Theory as a perspective to interpret these forms of crime committed by actors that are neither fully human nor fully machine. The two authors argue that it is not possible to understand the nature of crimes committed through botnets if we continue to use an anthropocentric criminology that sees humans as the primary driver of criminal behavior: *«[r]ather than considering a botnet as a technological tool, an individual (opportunistic) crime or an asset on the criminal market, we treat a botnet as a hybrid criminal network, a crime that results from human/technology mutual cooperation and interaction*» (van der Wagen and Pieters 2015: 579). The authors refer to human–machine networks, which can be backed by a variety of people and machines. This leads to the consideration that we might suspect the existence of completely new forms of “hybrid organized crime” committed by actants and that we should derive consequences on a theoretical level. Van der Wagen and Pieters’ is an interesting interpretive framework for modern forms of crime where the digital is a central element and the interaction between humans and non-humans is so profound that we need to think of a specific, digital typology of organized criminal networks. Actor-Network Theory is a sociological approach that may also prove useful in interpreting recent forms of human–machine interaction committed with and through artificial intelligence in organized crime (analyzed in Organized criminal activities in the digital society section).

We do not have to get as far as these forms of organized crime between human beings and machines, which seem almost like science fiction, but which we nevertheless increasingly have to deal with, to recognize that very different organized crime groups coexist and cooperate in the digital society, with different organizational models operating online and offline/online, relying on technology for different purposes, and interacting with computers in different ways, increasingly blurring the distinction between the physical and the virtual and technological dimensions of organized crime. New technologies are spawning new organized crime groups, changing the characteristics of old organized crime groups, and being exploited by organized crime groups that, while not changing their structures, are changing their activities. For this reason, there is a need for more elastic criminological interpretations, for concepts that do not separate the “virtual” from the “physical” dimension of organized crime, and organized cybercrime from organized crime (whether traditional or not) in a way that bears little resemblance to reality, as if they were opposing concepts that have nothing in common, without a lowest common denominator. All the “modern” modalities of organized crime that the digital society has brought should be fully contained in an updated concept of organized crime, and not beyond its reach. It could thus be argued that it is precisely the criminological definition of organized crime that needs to be revised today, especially in light of the new global phenomena brought about by digitalization.

## Digital organized crime and the spectrum theory of digital organized crimes

Today, it is increasingly difficult to separate (many) criminals who operate offline from those who operate online, and (many) crimes committed in the cyber world from those committed in the physical world. The two worlds are increasingly intertwined. In the digital society, people’s daily behaviors and physical habits are changing in response to the digital. The digital impacts the social dimension, online and offline. In the digital society, human and non-human actors interact and influence each other. This is also true for crime, which is nothing more than a subset of social behaviors practiced by certain social actors, the criminals, whose actions are increasingly socio-material, just like those of conforming actors. As we have seen, this is also true of organized crime. Therefore, today, in relation to organized crime, there is a need for a criminological definition that can take into account this complexity, this continuum of organized crimes and organized criminals between offline and online, this strong influence of new technologies on organized crimes (and their organization) and organized criminals (and their organization). There is no longer a need for a narrow definition based on partially obsolete paradigms, but for broad reference points, for a more modern theoretical approach capable of capturing the radical changes that digital society brings also in terms of organized crime, and for more modern research hypotheses: a theoretical approach to digital organized crime that allows us to consider the multiple facets of organized crime in digital society without abandoning both the technological category of organized cybercrime and the concepts of traditional organized crime that researchers have arrived at. In other words, one should take an approach of sociology of crime that attempts to track modern organized crime phenomena in the context of such a pervasive digital society.

The use of the term “digital organized crime” is proposed here not as a synonym for organized cybercrime, but as a concept that aims to capture the many manifestations of organized crime groups and organized crimes in digital society, including online organized cybercrime groups and online organized cybercrimes (with their undeniable specificity), which would represent the most technical subset. This concept makes it possible to interpret all forms of organized crime in its subjective and objective dimension, which takes shape in the modern digital society.

“Digital organized crime” refers to groups of individuals (and of individuals and machines, of actants) working together with different forms of specialization and professionalism and organizing in different ways to commit criminal activities online, offline/online, and offline that require organization of criminal work over a long, extended period of time and that are strongly influenced by the digital dimension in terms of the criminal activities they commit and the way these activities are organized, the organization of the criminal group itself, and the relationships with other criminal groups and criminal actors and machines. Within this concept, it is possible to integrate both online organized cybercrime groups and traditional organized crime groups, both groups of human actors and networks of human and non-human actors and the socio-material objects they produce in their interaction (networks of actants). The theoretical framework into which we propose to insert this definition is that of a current, revised sociological theory of the organization of crime and deviance in digital society, a new theory of digital criminal organizing (to be developed starting from the contributions of Cressey 1972; McIntosh 1975; Best and Luckenbill 1982; Rostami 2016; Rostami et al. 2018).

To better delineate the content of digital organized crime from the objective view of criminal behavior, the “spectrum theory of digital organized crimes” can be used: that is, any behavior of ‘modern’ organized crime can be positioned on a spectrum that is often a mixture of online and offline, ranging from completely technological and virtual organized crime behavior, fully realized in digital territories and which would not even exist without ICT, on the one hand, to completely physical organized crime behavior, without any contact with the digital world, on the other. Human–machine interactions and the construction of digital socio-material objects vary according to their position on this spectrum. And the concepts of space and time can also vary along the spectrum, as the more crime moves into the virtual dimension, the less space can have meaning and the more time can contract and be fast. The level of digital awareness of organized crime actors can also vary widely. Between these two extremes there are a variety of modes of adaptation, of nuances in the use of the digital by organized crime groups for their activities. Thus, by the term digital organized crimes we are not referring to organized cybercrimes, but to the organized crimes that take shape in the digital society along this continuum.

Regarding digital organized crime and the spectrum of digital organized crimes, we can learn much from the Covid-19 pandemic that has affected every aspect of our lives (see, e.g., Kemp et al. 2021). In the face of a general and decisive decrease in crimes characterized by physical aspects, the years of the pandemic (2020 and 2021) have seen a sharp increase in crimes related to the online world in all developed countries. As for the European Union, the crimes that have increased the most are not only cybercrime in the strict sense (such as ransomware or DoS or DDoS attacks), but also online theft and fraud related to digital identity, online fraud of all kinds, credit card cloning, and credit card fraud (Europol 2020). In general, online financial crime has increased, as has online sexual abuse of minors. Online counterfeiting and, more generally, online trafficking of illegal products, such as the sale of drugs, have also increased significantly (ibid.). In addition, online technologies and social interactions have played a central role in activities such as migrant smuggling, drug trafficking, and terrorism (Sanchez and Achilli 2020; Basit 2020). During the pandemic, migrant smugglers took advantage of the health crisis to attract more customers and raise prices (justifying this with the greater risks due to the pandemic), and increasingly did so by advertising their online services (Adhoob 2021). In the drug trade, communication between criminals via social media was one of the most important elements during the health crisis (Namli 2021). Unlike before the pandemic, when terrorist groups spread their beliefs and found new recruits through gatherings and meetings, in 2020 the Internet was the only way for extremists to reach new followers.

In other words, the global health crisis has triggered a “pandemic digital crime,” organized and unorganized, in the face of an overall decline in physical crime. We have thus been spectators of a natural experiment that, after the general lockdowns and near-absolute restrictions on travel and physical socialization in many countries, has shown us that today, in the world of organized and unorganized crime (as well as in the world of legality), the boundaries between offline and online are increasingly surmountable. Both individuals and criminal groups that have already engaged in traditional criminal careers can expand their criminal reach by moving into the virtual. Just as in the legal world, where even people who are not particularly tech-savvy have definitely expanded the use of digital technology in their normal daily routines, the same is true for organized and unorganized criminals who are not necessarily very tech-savvy. The pandemic has shown that there is the possibility of a digital shift of organized and unorganized crime, and therefore of perpetrators, from the physical to the virtual dimension, which is relatively easy for some crimes.

The lesson offered by the natural experiment of the pandemic is that criminals can easily reorganize themselves by moving from offline crime to online crime, or better yet, by integrating offline and online. The Covid-19 pandemic has interacted with the acceleration of the digital dimension of society like no other global event, including from the perspective of organized crime: the result has been that more and more crime, especially organized crime, has moved wholly or partially to the virtual and to the extreme edge of the digital spectrum, which is characterized by the intangible and technological dimension.

## Towards a digital sociology of organized crime

As in sociology (Lupton 2015; Marres 2017) and other disciplines such as anthropology (Horst and Miller 2013), the humanities (Berry 2012), and geography (Ash et al. 2018), digital criminology has recently emerged from the confluence of criminology and the digital with its profound social changes (Stratton et al. 2016; Smith et al. 2017; Powell et al. 2018; Di Nicola 2021). As we turn to organized crime in digital society, its study will increasingly require a digital sociology of organized crime.

Doing a digital sociology of organized crime today means, among other things, being interested in digital organized crime, perpetrators and victims of digital organized crime; social reactions to (digital) organized crime in digital society, and criminological research on (digital) organized crime in digital society, that is, social research that uses technology as a source of information about (digital) organized crime (digital data) and as a method for studying (digital) organized crime. A digital sociology of organized crime today means examining the opportunities and risks of the digital in the field of justice and crime prevention with reference to (digital) organized crime. The digital sociology of organized crime must also examine the effects (including potentially negative effects) that digital society may have on (digital) organized crime prevention and justice practices, on the representation of (digital) organized crime and on the construction of associated fears by the digital, and on the risks of a surveillance society, inequalities, and discriminatory practices related to (digital) organized crime.

Doing a digital sociology of organized crime means going beyond the paradigm of cybercriminology (Jaishankar 2018; Diamond and Bachmann 2015; Maras 2016; Jahankhani 2018; Moise 2020) applied to organized cybercrime without diminishing it in any way. The new digital sociology of organized crime is not cybercriminology interested in organized cybercrime, but it contains it because it is a broader and transversal framework. The interest in the digital sociology of organized crime is not only to study organized cybercrime, but also to study the impact of digital society on organized crime and on the digital, formal and informal, responses to (digital) organized crime, such as the use of artificial intelligence and robotics against (digital) organized crime (see, e.g., Interpol and Unicri 2019) or online crowdsourcing as a source of information gathering against (digital) organized crime to conduct investigations (see, e.g., Schneider and Trottier 2012) or Internet vigilantism (see, e.g., Nhan et al. 2017; Trottier 2017).

The digital sociology of organized crime will need to attempt to develop new concepts and new criminological theoretical paradigms about digital organized crime and the social response to it. It will need to test old criminological theories about organized crime in digital domains and develop and test new criminological explanations for the causes of digital organized crime. It will need to develop and test new ideas about the organization of organized crimes and organized criminals in digital society.

Doing a digital sociology of organized crime also means, and increasingly means, taking an interdisciplinary and multidisciplinary approach to security and crime research that brings criminology and the social sciences into deep dialog with many other scientific disciplines, including law, statistics, mathematics, computer science, engineering, economics, and cognitive science, to better understand and respond to organized crime in digital society. In the field we are talking about here, computer scientists and technology experts and even practitioners often take precedence over criminologists, meaning that technology and security studies and interventions often involve “a lot” of technology but “very little” criminological and social theory and the ability to examine ethical issues. Similarly, criminologists studying the digital dimension of organized crime and countering digital organized crime are unlikely to achieve meaningful results without the intent to engage with other disciplines.

Organized crime is a form of crime that is particularly sensitive to digitization because of its unique characteristics: this article calls on the scholarly community to do more to shape the frontiers of a new digital sociology of organized crime.
